# Determination of soluble solids content of multiple varieties of tomatoes by full transmission visible-near infrared spectroscopy

**DOI:** 10.3389/fpls.2024.1324753

**Published:** 2024-01-23

**Authors:** Sheng Li, Jiangbo Li, Qingyan Wang, Ruiyao Shi, Xuhai Yang, Qian Zhang

**Affiliations:** ^1^College of Mechanical and Electrical Engineering, Shihezi University, Shihezi, China; ^2^Intelligent Equipment Research Center, Beijing Academy of Agriculture and Forestry Sciences, Beijing, China; ^3^Xinjiang Production and Construction Corps Key Laboratory of Modern Agricultural Machinery, Shihezi, China; ^4^Key Laboratory of Northwest Agricultural Equipment, Ministry of Agriculture and Rural Affairs, Shihezi, China; ^5^Engineering Research Center for Production Mechanization of Oasis Characteristic Cash Crop, Ministry of Education, Shihezi, China

**Keywords:** tomato, soluble solids content, online detection, full transmission, quantitative analysis model

## Abstract

**Introduction:**

Soluble solids content (SSC) is a pivotal parameter for assessing tomato quality. Traditional measurement methods are both destructive and time-consuming.

**Methods:**

To enhance accuracy and efficiency in SSC assessment, this study employs full transmission visible and near-infrared (Vis-NIR) spectroscopy and multi-point spectral data collection techniques to quantitatively analyze SSC in two tomato varieties (‘Provence’ and ‘Jingcai No.8’ tomatoes). Preprocessing of the multi-point spectra is carried out using a weighted averaging approach, aimed at noise reduction, signal-to-noise ratio improvement, and overall data quality enhancement. Taking into account the potential influence of various detection orientations and preprocessing methods on model outcomes, we investigate the combination of partial least squares regression (PLSR) with two orientations (O1 and O2) and two preprocessing techniques (Savitzky-Golay smoothing (SG) and Standard Normal Variate transformation (SNV)) in the development of SSC prediction models.

**Results:**

The model achieved the best results in the O2 orientation and SNV pretreatment as follows: ‘Provence’ tomato (Rp = 0.81, RMSEP = 0.69°Brix) and ‘Jingcai No.8’ tomatoes (Rp = 0.84, RMSEP = 0.64°Brix). To further optimize the model, characteristic wavelength selection is introduced through Least Angle Regression (LARS) with L1 and L2 regularization. Notably, when λ=0.004, LARS-L1 produces superior results (‘Provence’ tomato: Rp = 0.95, RMSEP = 0.35°Brix; ‘Jingcai No.8’ tomato: Rp = 0.96, RMSEP = 0.33°Brix).

**Discussion:**

This study underscores the effectiveness of full transmission Vis-NIR spectroscopy in predicting SSC in different tomato varieties, offering a viable method for accurate and swift SSC assessment in tomatoes.

## 1 Introduction

Tomatoes, ubiquitous in global agricultural production, exhibit noteworthy nutritional significance (Passam et al., 2007). As an esteemed vegetable within the realm of human dietary practices, tomatoes assume a pivotal role (Zhu et al., 2015). They bestow a diverse complement of indispensable organic compounds, thus exerting a multifaceted influence encompassing pigmentation modulation, retardation of aging processes, lipid and blood pressure reduction, safeguarding of prostatic health, as well as reinforcement of gastric and hepatic functions (Perveen et al., 2015; Youssef and Eissa, 2017; Salehi et al., 2019). By virtue of their unique attributes, encompassing edibility, health-enhancing properties, and therapeutic potential, tomatoes and their derivative products occupy a prominent and indispensable position within the global landscape of agricultural production and trade (Guan et al., 2018; Ali et al., 2020). Soluble solids content (SSC) represents a pivotal constituent of tomato flavor, holding the potential to align closely with consumers’ perception of intrinsic quality attributes in tomatoes (Ponce-Valadez et al., 2016). Nevertheless, the conventional analytical methodologies employed for quantifying this quality parameter are beset with challenges related to protracted analysis durations, substantial costs, and environmental contamination (Skolik et al., 2019). The imperative for the tomato production and distribution industry, therefore, resides in the development of expeditious, facile, cost-effective, environmentally benign, and non-invasive techniques for batch quality control assessment, with the ability to extend precision down to the level of individual fruits (Najjar and Abu-Khalaf, 2021).

In the past few decades, many non-destructive testing techniques have been used to detect tomato SSC (Mei and Li, 2023). Gómez et al. (2008) used PEN 2 electronic nose (E-nose) to detect tomatoes with different storage time. The correlation between the measured value and the predicted value showed that the effect of using E-nose sensor signal to predict tomato SSC was poor. Nikbakht et al. (2011) used raman spectroscopy to determine the SSC of tomato. The root mean square error of predictions (RMSEP) of SSC measured by partial least squares regression (PLSR) and principal component regression (PCR) models were 0.30 and 0.38, respectively. In order to explore the possibility of mid-infrared spectroscopy for tomato quality detection, Ścibisz et al. (2011) used the attenuated total reflection accessory of the fourier transform spectrometer to scan the tomato samples in the wavenumber region of 4000 to 400 cm^-1^. The PLSR model has a reasonable ability to estimate the SSC of tomatoes, with a high determination coefficient (0.98) and a small prediction error (3%). Mollazade et al. (2015) used backscattering and multispectral imaging techniques to predict the quality factors of tomato fruit during storage. The correlation coefficients between the prediction results of SSC correction model established by artificial neural network and the reference measurement results of multispectral and backscatter imaging are 0.736 and 0.561, respectively. Rahman et al. (2017) established a non-destructive method for the determination of SSC in intact tomatoes using hyperspectral imaging technology in the range of 1000-1550 nm. The PLSR model based on smoothing pretreatment spectrum has a good prediction effect on SSC of intact tomatoes, with a correlation coefficient of prediction (Rp) of 0.74 and a RMSEP of 0.33%.

While the aforementioned methods enable non-destructive testing, their inherent time-consuming nature and elevated cost factors constrain their utility when catering to the rigorous industrial testing requisites characteristic of large-scale tomato production. In stark contrast, the visible and near-infrared (Vis-NIR) spectroscopy technique emerges as an expedient solution (a non-destructive, expeditious, real-time, and cost-effective approach) to effectuate internal quality appraisal within the domain of agricultural product evaluation. Torres et al. (2015) used NIR reflectance spectroscopy to determine the SSC of Raf tomato based on modified PLSR (coefficient of determination for cross-validation is 0.75; standard error of prediction is 0.65%). Acharya et al. (2017) conducted a practical evaluation in the context of the non-destructive determination of the dry matter content of intact tomatoes (an indicator of the final mature SSC) using a handheld visible-short-wave NIR spectrophotometer. By using populations with different harvest dates or growth conditions for calibration and prediction, the dry matter prediction coefficient of determination (R^2^) is 0.86-0.92, and the deviation is 0.14-0.03%. At different maturity stages of specific tomato varieties, Zhang et al. (2021) reported the acceptable prediction results of SSC evaluation by the self-developed Vis-NIR portable system (Rp = 0.70, RMSEP = 0.26%) and NIR integrating sphere system (Rp = 0.82, RMSEP = 0.21%). Aiming at the characteristics of tomato internal heterogeneous structure, in order to obtain more internal information of tomato as much as possible, Wang et al. (2018) built a tomato Vis-NIR diffuse transmission detection system to detect the SSC of cherry tomato, showing good prediction results (Rp = 0.93, RMSEP = 0.36%). However, the typical Vis-NIR spectroscopy is limited to a small area of measurement, and the spatial information of the sample obtained by single point measurement is limited. Liu et al. (2019) developed a dynamic online sorting system based on Vis-NIR diffuse transmission, and the sorting accuracy of SSC reached 91%. Yang Y. et al. (2022) based on the Vis-NIR diffuse transmission system, optimized the detection settings such as light path and light intensity, and compensated the model according to the height and weight physiological traits of tomato samples, and achieved good results (Rp = 0.91, RMSEP = 0.17%). The limitations of traditional single-point Vis-NIR measurement technology can be overcome by using on-line full transmission measurement and continuous data acquisition. Compared with the diffuse transmission mode, the full transmission mode and tomato multi-point spectral measurement acquisition can achieve a comprehensive characterization of the entire tomato information.

The main purpose of this study is to determine the best model for SSC prediction of tomato based on full transmission Vis-NIR spectroscopy detection technology. The specific purposes are as follows: (1) Collecting Vis-NIR spectral data of all tomato samples using full transmission Vis-NIR online detection equipment; (2) Processing continuous multi-point spectral data using the weighted average method; (3) Establishing a PLSR model based on full-spectrum data, comparing the model’s performance, and selecting the optimal preprocessing method and the best detection orientation; (4) Applying the least angle regression method to extract characteristic wavelengths in tomato SSC detection, and determining the optimal prediction model by combining prediction accuracy and stability.

## 2 Materials and methods

### 2.1 Experimental samples

**Figure 1:**
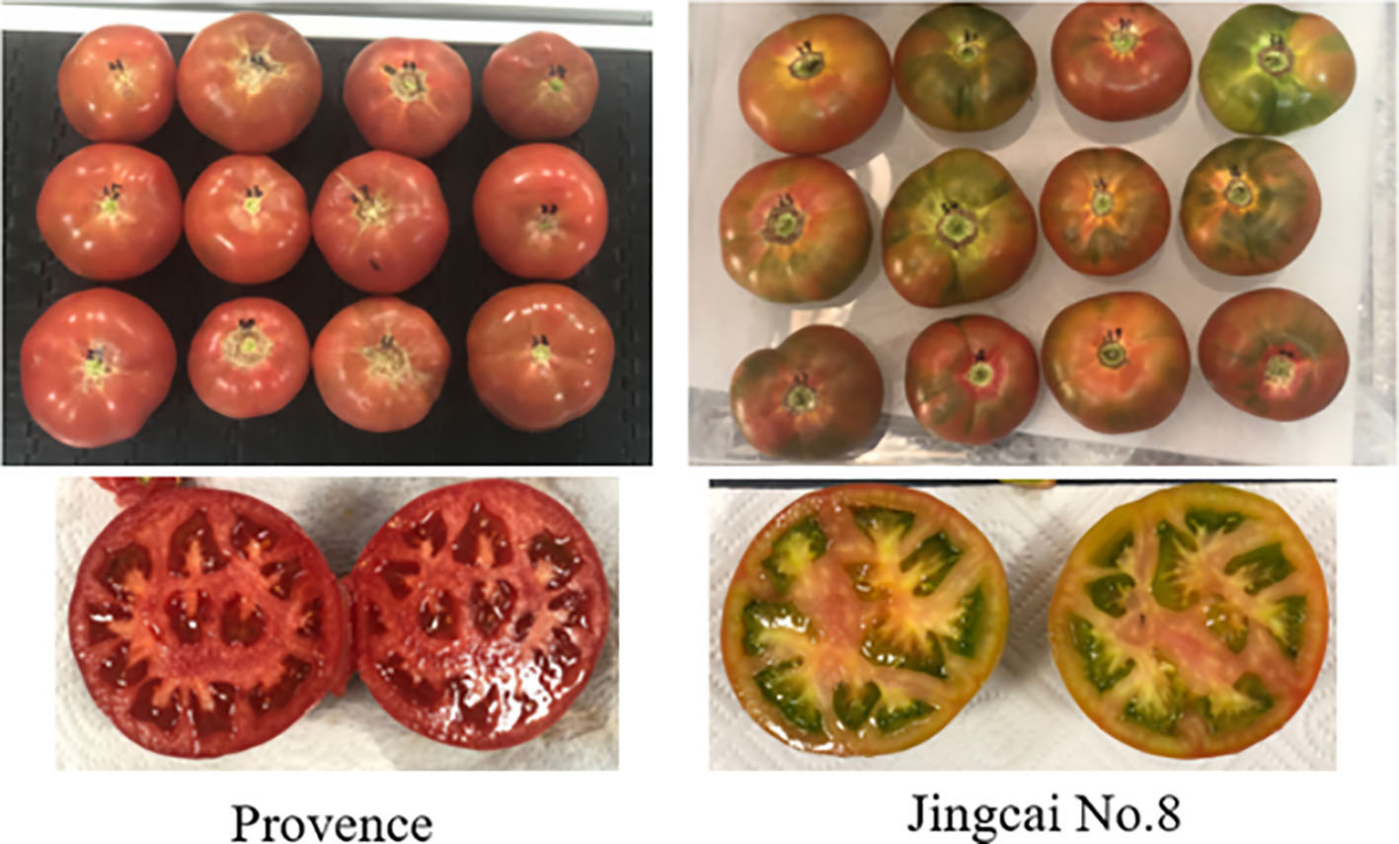
Tomato samples and cross sections.

In this study, we focused on two prominent tomato varieties, namely ‘Provence’ and ‘Jingcai No.8’ tomatoes, both of which enjoy substantial popularity in China. The ‘Provence’ tomato exhibits a thin skin, with succulent flesh, and attains a rich ruddy hue when reaching maturity. On the other hand, ‘Jingcai No.8’ tomato often referred to as strawberry tomato, features an orange-red or red peel with green shoulder, and its skin possesses a slight thickness. A comprehensive set of tomato samples encompassing various stages of maturity was meticulously collected to bolster the robustness of our predictive model for tomato SSC. These tomatoes were harvested from a farm located in the Fangshan District of Beijing, China. Tomato samples were collected from three maturity stages of half-ripe, hard-ripe and full-ripe, with a ratio of 1: 1: 1. The representative tomato samples obtained during this collection process are visually depicted in **Figure 1**. Moreover, in order to mitigate potential temperature-induced fluctuations that could influence the precision of our prediction model, the harvested tomatoes were transported to our laboratory facility and placed for a 24-hour period at a temperature of 20°C, with a relative humidity level of 60%, prior to the acquisition of spectral and SSC data (Yang X. et al., 2022).

To ensure the rigor and objectivity of our predictive model, we employed a systematic approach to partition all collected samples into two distinct subsets: a calibration set and a prediction set. This division was executed following a consistent ratio of 7:3, wherein 70% of the samples were allocated to the calibration set, responsible for the construction of the prediction model, while the remaining 30% constituted the prediction set, serving as an independent dataset for the assessment of model performance. And in order to mitigate the influence of random variability in sample partitioning and to provide a robust evaluation of our predictive model’s efficacy, we undertook a systematic randomization process. Specifically, we repeated the sample division procedure 100 times, each time generating a new partition of samples. Subsequently, we constructed a predictive model based on the results of each of these 100 divisions. The culmination of these 100 modeling outcomes was then leveraged to derive an average, which serves as the foundational basis for evaluating the overall performance of our predictive model (Tian et al., 2022). This approach ensures a comprehensive and reliable assessment of the model’s predictive capabilities.

### 2.2 Full transmission spectrum and real SSC acquisition

**Figure 2:**
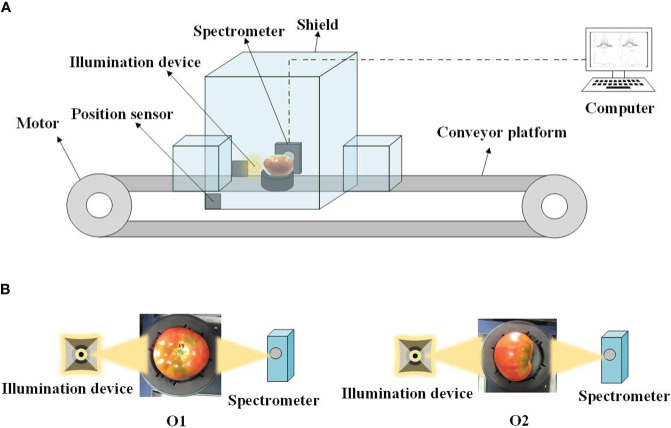
**(A)** Online full transmission spectroscopy and **(B)** detection orientations.

The full transmission Vis-NIR detection system, developed by the Intelligent Equipment Research Center of Beijing Academy of Agriculture and Forestry Sciences (Beijing, China), was used to acquire spectral data for all samples. This system, as depicted in **Figure 2A**, primarily comprises a highly sensitive spectrometer covering a wavelength range from 560 to 1072 nm and offering a spectral resolution of 0.5 nm. Furthermore, it is equipped with a conveyor platform featuring adjustable speed, a position sensor, and an illumination device consisting of a reflective halogen lamp (FUJI, JCR, 150W, 15V, Tokyo, Japan) with a focusing lens. The system is fortified with a shielding mechanism to prevent stray light interference and is under the control of a computer-based system. Both the illumination device and the spectrometer are positioned on opposite sides of the conveyor belt.

To assess the influence of spectral measurement orientation on the accuracy of online SSC detection in tomatoes, spectral data for all tomato samples were collected in two orientations known as O1 and O2. In the O1 orientation, the tested tomato’s stem-calyx axis was oriented perpendicular to the conveyor belt, with the stem facing upward. The sample received illumination from a halogen lamp at the equatorial position and was detected by the spectrometer on the opposite side. Conversely, in the O2 orientation, the stem-calyx axis of the tested tomato was parallel to the conveyor belt, with the stem directed towards the spectrometer. Schematic representations of these two detection orientations can be seen in **Figure 2B**. During each spectral measurement, the tomato sample was initially positioned on a fruit tray and moved at a consistent speed of 0.8 m/s. After the tomato sample passes through the sensor and the specified delay time, the spectrometer (integration time is 5ms) begins to continuously collect the spectral signals at each position on the sample. The multi-point spectra of each sample are roughly: ‘Provence’ tomato: 17-31 spectral curves; ‘Jingcai No.8’ tomato: 20-29 spectral curves.

Tomato SSC determination reference NY/T 2637-2014, using refractometer method, measuring instrument for digital Abbe refractometer. SSC measurements were performed immediately using the traditional method of destruction. Each complete tomato sample was first cut into pieces and squeezed into tomato juice in a wall-breaking machine. Then filter the tomato juice with gauze and squeeze into the beaker. After full shaking, the tomato juice was dropped on a hand-held digital refractometer, and the SSC value was manually recorded. Each measurement was repeated three times, and the average value was taken as the experimental value.

### 2.3 Data pre-processing

#### 2.3.1 Multi-point spectrum weighted average

When the online full transmission mode collects the spectral signal of the sample, the incomplete signal is collected at both ends, so they are first eliminated before further data processing. The use of a weighting method signifies an effective strategy for spectral analysis (Somers et al., 2011; Zhu et al., 2019). In this study, distinct weights are assigned to individual data acquisition locations based on either the signal-to-noise ratio (*SNR*) (quality assessment metric). The spectrometer is placed in an environment without the sample to be tested, and the spectral signal in the environment is recorded to obtain the background. The operational process can be outlined as follows:

Firstly, an evaluation of the *SNR* is performed, enabling the quantitative characterization of *SNR* for each data acquisition point. *SNR* serves as a quantitative metric for signal quality assessment. Following this, weight factors are calculated, with each data acquisition point being assigned a weight based on the *SNR*, also referred to as the quality assessment metric. This study employs an inverse relationship where the weight factor increases with a higher quality assessment metric. In conclusion, a weighted average is carried out, involving the multiplication of spectral data associated with each acquisition point by its respective weight factor. This shows the derivation process of the final effective spectral representation. Through the application of the weighting method, high-quality spectral data significantly impacts the final effective spectrum, while the influence of low-quality spectral data is minimized.

#### 2.3.2 Pretreatment of spectral data

To improve the correlation between tomato transmittance spectra and SSC and reduce the impact of unwanted signals and noise, it is customary to conduct preprocessing on the raw spectra. Among the common preprocessing methods, the application of Savitzky-Golay smoothing (SG) is instrumental in making spectral curves more conducive to the recognition of features and localization of peaks, thus enhancing precision (Zhao et al., 2022). Furthermore, the use of standard normal variable transformation (SNV) serves to emphasize the positions of spectral peaks, streamlining the analysis of both spectral shape and peak locations (Bázár et al., 2016). In this study’s context, two specific preprocessing techniques, a 13-point SG and SNV, have been implemented to refine the spectral data.

### 2.4 Prediction model and evaluation

An effective method for multivariate data analysis frequently employed in spectral analysis is PLSR. In this research, we constructed a PLSR model to delineate the quantitative relationship between the spectral matrix (**X**) and the matrix of SSC values in tomatoes (**Y**). To evaluate the mathematical approach’s performance, we utilized metrics such as the calibration correlation coefficient (Rc), root mean square error of calibration (RMSEC), prediction correlation coefficient (Rp), and root mean square error of prediction (RMSEP). The specific calculation formula can be seen in **Equations 1**, **2**. A robust model demonstrates correlation coefficients approaching 1 and lower root mean square error values (Li L. et al., 2022; Tian et al., 2023). In the process of model development, the optimal number of latent variables (LVs) is a critical consideration to prevent underfitting or overfitting issues (Diniz et al., 2015). To address this concern, we adopted a 5-fold cross-validation approach to determine the ideal number of LVs based on the minimum root mean square error of cross-validation (Li et al., 2023). The model was constructed using Matlab 2022b (Mathworks, Natick, MA).


(1) $$\begin{array}{ll} \text{RC} & ,\text{RP}=\sqrt{1-\frac{\sum_{i=1}^{n}{\left(y_{i}-{\hat{y}}_{i}\right)}^{2}}{\sum_{i=1}^{n}{\left(y_{i}-{\bar{y}}_{i}\right)}^{2}}} \end{array}$$

(2) $$\begin{array}{ll} \text{RMSEC} & ,\text{RMSEP}=\sqrt{\frac{1}{n}{\sum_{i=1}^{n}\left(y_{i}-{\hat{y}}_{i}\right)}^{2}} \end{array}$$

In this context, *y_i_* and ${\hat{y}}_{i}$ denote the measured value and the predicted value for the *i*th tomato sample within either the calibration or prediction dataset. Additionally, $\bar{y}$ represents the mean value of the measured values for samples within the calibration or prediction dataset, and *n* signifies the total number of samples in either the calibration or prediction dataset.

### 2.5 Wavelength selection methods

Within the domain of full spectrum modeling, a total of 2047 wavelengths are present, including a substantial number of irrelevant and collinear variables. These extraneous wavelengths, in addition to complicating the model, have the potential to introduce interference, which could result in a reduction in model accuracy (Luo et al., 2022). Thus, to address this, the least angle regression (LARS) technique was implemented for the purpose of identifying and selecting pertinent wavelengths.

Efron et al. (2004) introduced the LARS algorithm, a method that functions as a feature selection technique applicable to both linear regression and sparse regression. Its primary aim is to pinpoint features with strong correlations to the response variables (SSC) and to retain only these essential features within the model. This approach effectively simplifies the model, thus enhancing its capacity for generalization. LARS proceeds by incrementally integrating features and moving along the gradient direction of these features in each step. What sets LARS apart is its utilization of the regression coefficient path, allowing the simultaneous addition of multiple features. At the core of LARS is the consistent alignment with the prevailing gradient direction at each step, coupled with the allocation of suitable step sizes between features. This approach enables LARS to promptly and reliably identify characteristic wavelengths highly correlated with SSC. Nonetheless, LARS may face efficiency challenges when handling extensive feature sets. To tackle this limitation, the present study introduces regularization terms in the form of the L1 norm (Lasso penalty) and L2 norm (Ridge penalty). The L1 norm penalty streamlines feature selection by reducing coefficients of irrelevant features to zero, resulting in the construction of a sparse model. This feature is particularly advantageous when dealing with high-dimensional data and problems involving the selection of essential wavelengths for SSC analysis. Conversely, the L2 norm penalty trims model parameters to prevent overfitting and enhance model generalization. Differing from L1 regularization, L2 regularization refrains from entirely zeroing out parameters, offering controlled adjustment of model complexity. This feature proves beneficial in addressing issues related to collinearity and augmenting model stability.

In this study, the specific regularization term is denoted by λ. A range of λ values is systematically selected, typically starting with a smaller value and gradually increasing it. Model performance is monitored, and an appropriate λ value is selected accordingly. Characteristic wavelength selection was carried out using Matlab 2022b (Mathworks, Natick, MA).

## 3 Results and discussion

### 3.1 SSC values of all samples

In this experiment, tomato samples with different maturity were collected to establish SSC prediction model. The SSC values of all samples measured are shown in **Table 1**. The SSC range of ‘Provence’ tomato is 3.8-8.7°Brix, and that of ‘Jingcai No.8’ tomato is 4.5-9.8°Brix. The standard deviations (SD) were 1.1 and 1.2°Brix, respectively. SSC has a wide range of distribution. Combined with the characteristics of different maturity samples, a more comprehensive and accurate SSC prediction model can be established. This method can enhance the robustness of the model and improve the accuracy of the prediction results (Li Y. et al., 2022). At the same time, it can also better cope with the different maturity of tomato samples that may occur in practical applications.

**Table 1:** SSC values (°Brix) of tomato samples.

Variety	Range	No. of samples	Mean	SD
Provence	3.8-8.7	92	5.8	1.1
Jingcai NO.8	4.5-9.8	96	7.4	1.2

The SSC values (°Brix) were measured by refractometer.

### 3.2 Analysis of tomato spectral feature

**Figure 3:**
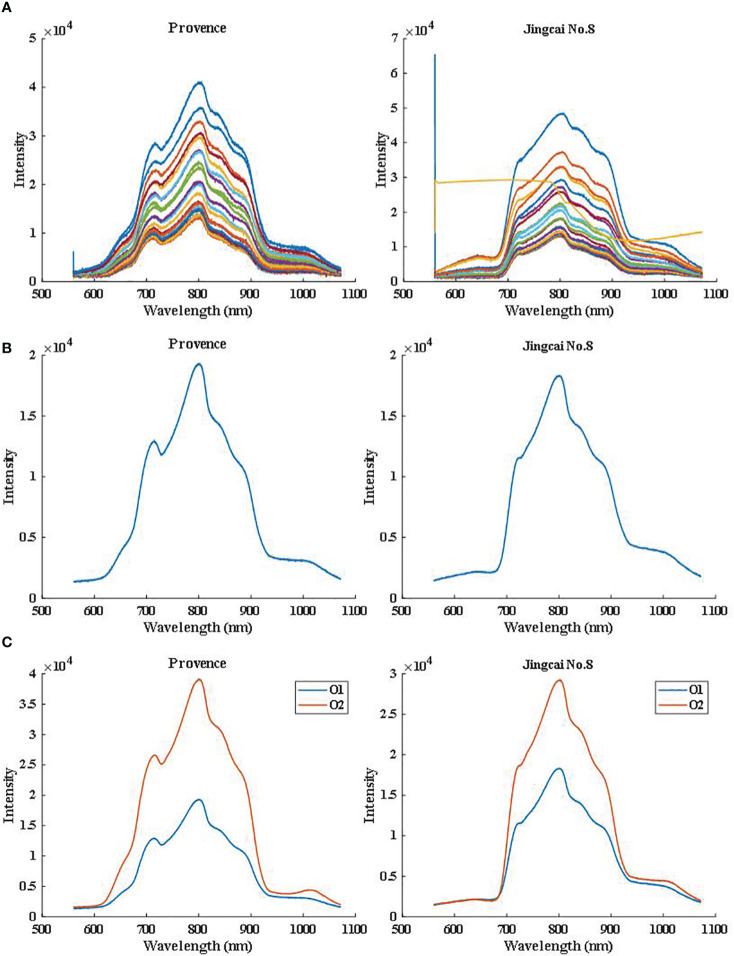
Spectral curve from **(A)** multi-point raw data, **(B)** weighted average and **(C)** two detection orientations.

**Figure 3A** shows that the multi-point spectral curves collected by each sample have problems such as low *SNR* and intensity supersaturation due to the acquisition method of online full transmission measurement, which may be caused by the texture color characteristics of tomatoes and the online acquisition method of spectrum. According to the spectral contribution of different parts, we can see the curve shown in **Figure 3B**. After the weighted average method, the noise in the data is effectively reduced, the *SNR* is improved, and the characteristic peak is enhanced, which is more conducive to the establishment of the subsequent prediction model. In **Figure 3C**, we can observe that the spectral curve characteristics of the same variety in different directions are basically similar. The main difference is the intensity, which may be due to the influence of the internal cavity structure of the sample on the propagation light path. Because the optical path of O2 orientation is simpler, the optical path distance is shorter, and the flesh tissue is less penetrated, the spectral curves of both varieties show that the intensity of O2 orientation is higher than that of O1.

### 3.3 Prediction of tomato SSC with full spectra

**Table 2** presents the outcomes derived from PLSR modeling applied to spectral data with the utilization of diverse preprocessing techniques. Notably, whether considering the ‘Provence’ tomato or ‘Jingcai No.8’ tomato, the results consistently manifest superior O2 performance as opposed to O1. This intriguing phenomenon is likely attributed to the inherent simplicity of the optical propagation pathway or the shortened propagation distance in the O2 direction. Conversely, the trajectory of incident light in the O1 direction necessitates traversal through discrete cavities, often yielding a more intricate optical pathway and extended propagation distances. In this study, the application of the SG method yields results marginally less favorable than the unprocessed data. This discrepancy could be ascribed to the potential obfuscation of essential spectral features by the SG method. Enhanced results are attainable through the adoption of preprocessing methodologies such as SNV. SNV preprocessing methods adeptly ameliorate scattering influences within the spectra, thereby endowing the data with increased stability, consistency, and diminished variability, consequently yielding a positive influence on modeling and quantitative analytical outcomes. The findings indicate that, subsequent to optimal preprocessing, the samples exhibit Rp = 0.81, RMSEP = 0.69°Brix (‘Provence’ tomato) and Rp = 0.84, RMSEP = 0.64°Brix (‘Jingcai No.8’ tomato). Obviously, the SNV preprocessing process magnifies the spectral attributes, making the spectrum clearer, more consistent and more prominent, thereby improving the quality of the spectral data set. In summary, this study established a robust PLSR model for SSC prediction. The model is founded on the determination of the optimal detection orientation (O2) in synergy with the implementation of the most effective preprocessing method (SNV).

**Table 2:** Prediction results of SSC of two varieties of tomatoes based on PLSR model established using full-spectrum data combined with different detection orientations and preprocessing methods.

Varieties	Orientations	Methods	LVs	Rc	RMSEC	Rp	RMSEP
Provence	O1	RAW	11	0.91	0.44	0.66	0.89
		SG	11	0.88	0.51	0.65	0.88
		SNV	9	0.95	0.31	0.70	0.84
	**O2**	RAW	10	0.89	0.50	0.73	0.82
		SG	11	0.88	0.52	0.74	0.80
		**SNV**	**9**	**0.94**	**0.36**	**0.81**	**0.69**
Jingcai NO.8	O1	RAW	9	0.94	0.39	0.62	1.04
		SG	11	0.89	0.53	0.62	1.10
		SNV	10	0.98	0.25	0.79	0.73
	**O2**	RAW	10	0.91	0.47	0.74	0.81
		SG	10	0.88	0.55	0.74	0.81
		**SNV**	**9**	**0.96**	**0.31**	**0.84**	**0.64**

### 3.4 Determination of the optimal model

While the full-spectrum PLSR model can effectively predict SSC quantitatively, most full-spectrum models exhibit instability due to notable disparities between Rc and Rp. Given that an excessive number of spectral variables employed in modeling can lead to overfitting, this study implemented characteristic wavelength selection to optimize the model. Through the selection of wavelengths, superfluous features are reduced, rendering the model more concise and efficient. Since O2 represents the optimal detection orientation, and SNV serves as the most effective preprocessing method, variable selection was exclusively based on the spectral data acquired in the O2 orientation and after preprocessing using SNV.

During the deployment of the LARS method for characteristic wavelength selection, initialization is initiated at the outset. The model begins by not selecting any characteristic wavelengths, with all coefficients set to zero. Subsequently, in each step, the system identifies the characteristic wavelength displaying the highest correlation with SSC and calculates the absolute value of this correlation. The selection of characteristic wavelengths follows a path along the minimum angle. Then, the coefficients are updated, with the coefficient of the selected characteristic wavelength gradually increasing until its correlation with another characteristic wavelength equals it. The regularization parameter λ is then progressively adjusted to achieve a balance between characteristic wavelength selection and model sparsity. Ultimately, the steps following the initializations are reiterated until the selection outcome converges.

**Figure 4:**
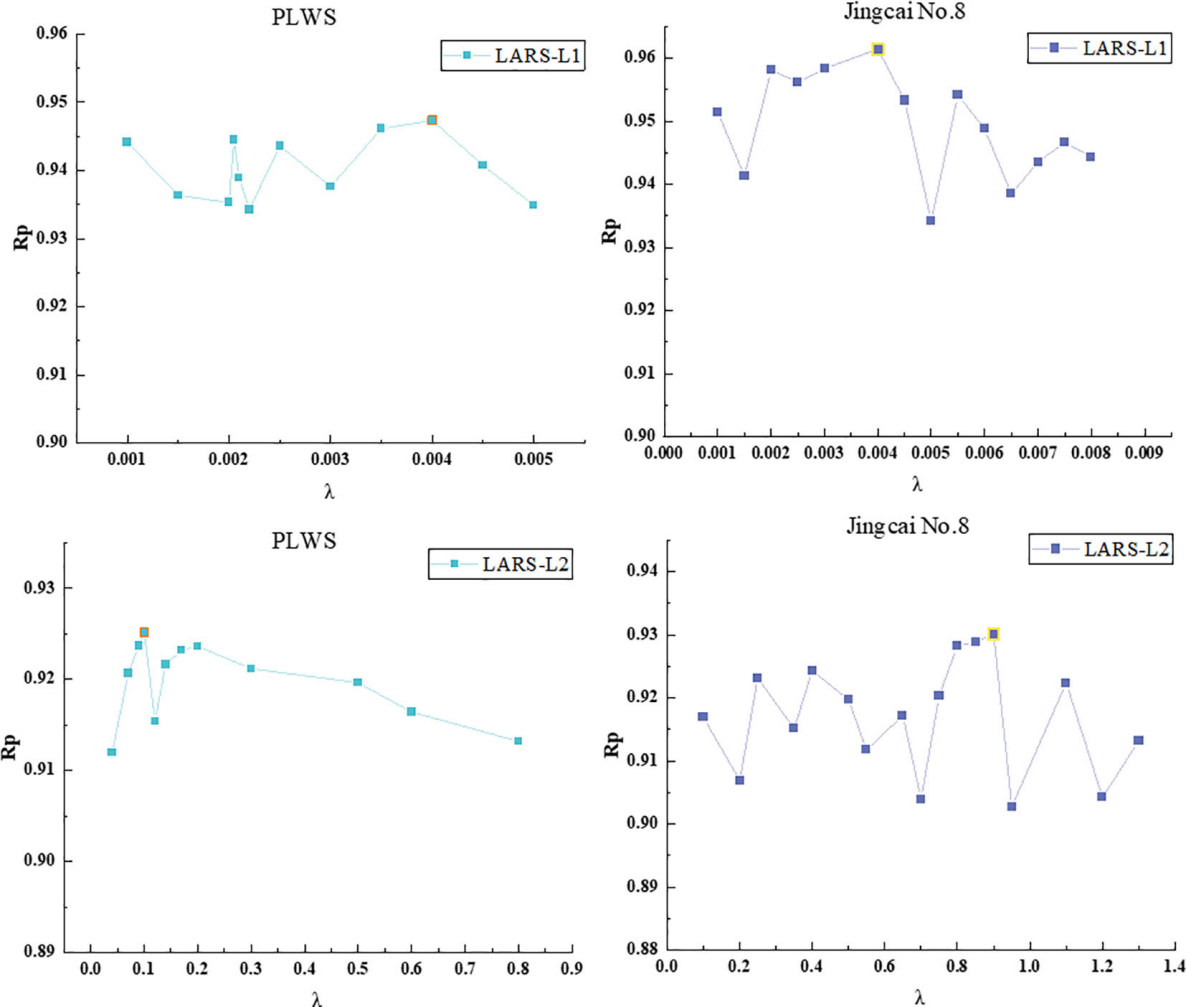
The process of Rp changing with λ in the process of characteristic wavelength selection.

A series of selected λ values and the corresponding Rp relationship are shown in **Figure 4**. It can be seen that LARS-L1 and LARS-L2 are significantly different in the selection range of λ, which may be due to the encouragement of L1 regularization to sparsity: L1 regularization encourages sparsity by punishing the absolute value of the coefficient, that is, encouraging the model to reduce the coefficient of most features to zero, so as to select the most important features, which usually requires a relatively small λ to achieve. L2 regularization encourages smoothness: L2 regularization reduces the magnitude of the coefficients by penalizing the square of the coefficients, thereby encouraging the coefficients of the feature to be evenly distributed, but it does not compress the coefficients to zero. Therefore, in order to achieve an effective L2 regularization effect, a relatively large λ is usually required. Although there are some fluctuations in the model Rp results obtained under different λ values, the changes are also around 0.01-0.03, and the results are relatively good (Rp > 0.90).

In the prediction model results of LARS-L1 method, the Rp of ‘Provence’ tomato and ‘Jingcai No.8’ tomato were both above 0.93. When the regularization parameter λ = 0.004, the effect was the best (‘Provence’ tomato: Rc = 0.98, RMSEC = 0.23°Brix, Rp = 0.95, RMSEP = 0.35°Brix; ‘Jingcai No.8’ tomato: Rc = 0.98, RMSEC = 0.20°Brix, Rp = 0.96, RMSEP = 0.33°Brix). L1 regularization tends to generate sparse solutions, so that some weights are zero, and a small number of characteristic wavelengths that have a significant impact on SSC prediction can be selected to filter out wavelengths that are not important for prediction. The best performance of the LARS-L1 method is due to its sparseness of L1 regularization and better capture of the correlation between wavelength and SSC. LARS-L2 obtained the best effect of ‘Provence’ tomato at λ = 0.1 (Rc = 0.97, RMSEC = 0.26°Brix, Rp = 0.93, RMSEP = 0.42°Brix). ‘Jingcai No.8’ tomato achieved the best results at λ = 0.9 (Rc = 0.97, RMSEC = 0.28°Brix, Rp = 0.93, RMSEP = 0.45°Brix). The penalty of L2 regularization on feature weights is balanced, and the weight of ownership is relatively evenly reduced without deleting some features too much. When there are some relatively weak wavelengths in the data that still contribute to the prediction, L2 regularization preserves these wavelengths. This may be the reason that the performance of LARS-L2 is slightly lower than that of LARS-L1. The LARS-L1 and LARS-L2 methods have less influence on the correlation of features because they constrain the feature weights through regularization and exhibit a degree of stability. The best results of the two characteristic wavelength selection methods for the two varieties were placed in **Table 3**.

**Table 3:** SSC prediction results obtained by characteristic wavelength PLSR models.

Varieties	Wavelength selection methods	λ	LVs	No. of variables	Rc	RMSEC	Rp	RMSEP
Provence	**LARS-L1**	**0.004**	**9**	**29**	**0.98**	**0.23**	**0.95**	**0.35**
	LARS-L2	0.1	10	53	0.97	0.26	0.93	0.42
Jingcai NO.8	**LARS-L1**	**0.004**	**9**	**63**	**0.98**	**0.20**	**0.96**	**0.33**
	LARS-L2	0.9	11	45	0.97	0.28	0.93	0.45

**Figure 5:**
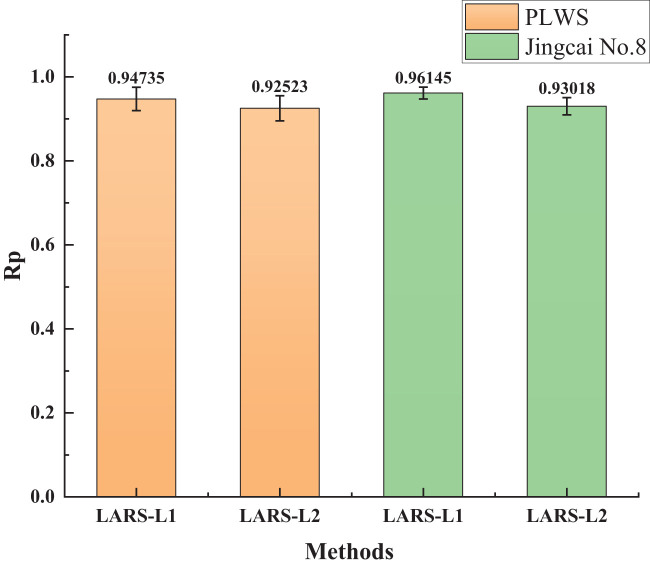
The error bar graph of 100 iterations under optimal λ.

In practical applications, aside from predictive accuracy, model stability is also a crucial consideration. **Figure 5** illustrates the error bar chart for the prediction results of SSC for two tomato varieties based on 100 modeling iterations, incorporating the optimal feature wavelength selection from two methods. Each data point in the figure is associated with an error bar, and the central mark denotes the mean value, reflecting the data’s central tendency. These error bars signify the data’s dispersion or uncertainty. It is evident that the results of both methods align with the trends depicted in **Figure 4**. The LARS-L1 method exhibits the highest mean Rp value, consistent with the central tendency of the data, and features shorter error bars, indicative of lower dispersion. Therefore, overall, the model demonstrates a heightened level of stability.

## 4 Conclusions

In this study, the SSC of tomato was successfully determined using the full transmission Vis-NIR spectroscopy online detection equipment. The results show that the weighted average method can improve the spectral quality of multi-point spectral data. The prediction performance of O2 is better than that of O1 in the detection orientation, and the prediction performance of the full-spectrum PLSR model constructed after SNV pretreatment is significantly improved. For the samples in the prediction set, the results of the two varieties of tomatoes were ‘Provence’ tomato: Rp = 0.81, RMSEP = 0.69°Brix; ‘Jingcai No.8’ tomato: Rp = 0.84, RMSEP = 0.64°Brix. In addition, in order to reduce the number of variables involved in modeling, the LARS method combined with L1 and L2 regularization is used to select the characteristic wavelengths to construct the PLSR model. The results show that the prediction accuracy of the characteristic wavelength selection model is better than that of the full spectrum model. When λ was set to 0.004, the characteristic wavelengths selected by the LARS-L1 method achieved the best results on the SSC prediction models of the two varieties of tomatoes (‘Provence’ tomato: Rp = 0.95, RMSEP = 0.35°Brix; ‘Jingcai No.8’ tomato: Rp = 0.96, RMSEP = 0.33°Brix). Under the condition of optimal λ, 100 modeling calculations were carried out to further verify the stability of the model. Finally, O2-SNV-LARS-L1-PLSR was determined as the best model for quantitative detection of tomato SSC, and it showed that this method combined with full transmission Vis-NIR spectroscopy had the potential for non-destructive detection of SSC in tomato.

## Data availability statement

The raw data supporting the conclusions of this article will be made available by the authors, without undue reservation.

## Ethics statement

This article has no any study with human participants or animals by any of the authors.

## Author contributions

SL: Writing – original draft. JL: Writing – original draft. QW: Writing – review & editing. RS: Writing – review & editing. XY: Funding acquisition, Writing – review & editing. QZ: Supervision, Writing – review & editing.
